# Paneth cell metaplasia in newly diagnosed inflammatory bowel disease in children

**DOI:** 10.1186/1471-230X-14-93

**Published:** 2014-05-15

**Authors:** Naomi Simmonds, Mark Furman, Evi Karanika, Alan Phillips, Alan WH Bates

**Affiliations:** 1 Tissue Sciences, St Thomas’s Hospital, London SE1 7EH, UK; 2 Child Health Department, Royal Free Hospital, London NW3 2QG, UK

**Keywords:** Paneth cell, Metaplasia, Paediatric, Ulcerative colitis, Crohn’s disease

## Abstract

**Background:**

Paneth cell metaplasia (PCM) is well described in adults but little is known about the distribution of colonic Paneth cells and the occurrence of PCM in a paediatric population. The aim of this study is to determine whether Paneth cell hyperplasia or metaplasia characteristically occurs in the colons of children with newly diagnosed idiopathic inflammatory bowel disease (IBD).

**Methods:**

We retrospectively reviewed colonic series from 28 new diagnoses of paediatric IBD at a tertiary referral centre, and from a further 14 children with IBD-like symptoms whose colonic biopsies and ancillary investigations were normal. Paneth cells were counted at 6 anatomical sites in the colon, and at each site acute and chronic inflammation were assessed semi-quantitatively and the presence or absence of crypt architectural distortion and eosinophilia was documented.

**Results:**

In control, ulcerative colitis (UC) and Crohn’s disease (CD) groups there was a gradient of decreasing Paneth cell numbers from caecum to rectum. Paneth cells were not seen in the distal colon in the control group, but they were present there in 11 of 13 patients with ulcerative colitis and 14 of 15 with Crohn’s disease. Only patients with IBD showed Paneth cell hyperplasia, assessed as more than 10 Paneth cells per 10 well-oriented crypts at any site. There was a statistically significant increase in Paneth cells in the caecum, ascending, transverse and descending colon in UC and in the ascending, transverse, descending and sigmoid colon in CD compared with controls. There was no significant difference between UC and CD. There was no correlation between the site of PCM and acute or chronic inflammation, crypt distortion or eosinophilia.

**Conclusion:**

Paneth cells are found in the proximal but not the distal colon in otherwise normal paediatric colonic series. A high proportion of UC and CD patients show PCM in the distal colon. This is present early in the disease and does not correlate with histological features of chronicity.

## Background

Paneth cells, named after Joseph Paneth [1], are unique epithelial cells responsible for secreting the antimicrobial alpha defensin peptides HD5 and HD6 as well as enzymes including lysozyme and phospholipase A2 that act to keep the intestinal crypts sterile [2-7]. They play a key role in defense against a broad range of intestinal microbes and in the regulation of host immunity [8]. Paneth cells derive from fast-cycling crypt base columnar cells, which are involved in crypt regeneration; they differentiate whilst migrating towards the crypt base, from which they are eventually cleared by phagocytosis [9,10]. Paneth cells are mostly located within the small bowel; in adults they occur normally in smaller numbers in the proximal large bowel (caecum to transverse colon), but have been reported more distally only in pathological states [11].

Paneth cells are termed ‘metaplastic’ when seen in areas in which they are not normally found: Paneth cells in the distal colon (descending colon, sigmoid and rectum) are always metaplastic. In pathological states, an increase in Paneth cell numbers – known as Paneth cell hyperplasia – may occur in the proximal colon [11]. Paneth cell metaplasia (PCM) has been most often described in idiopathic inflammatory bowel disease (IBD), both ulcerative colitis (UC) and Crohn’s disease (CD). In adults, it is thought to be a sign of a long colitis history: it correlates with disease duration, and it has been attributed to the effects of repair and regeneration [11-13]. Guidelines for reporting gastrointestinal biopsies published by the British Society of Gastroenterology in 1997 [14] concurred that PCM was an indicator of chronic epithelial cell damage, though it was not included in the data set for IBD reporting as its diagnostic value was unclear. PCM may be present in other pathological states – in neonates, Paneth cell numbers are increased in the regenerating bowel following necrotizing enterocolitis [15] – but is not seen in self-limiting infectious colitides [16,17].

There have been few studies of PCM in paediatric populations. IBD may present differently in this age group: new presentations of ulcerative colitis in children show a different pattern of inflammation from that seen in adults [18]. In this study, we describe the distribution of Paneth cells in symptomatic children without significant gastrointestinal pathology, compare the findings with newly diagnosed IBD, and evaluate the relation between PCM and histological features of chronic disease.

## Methods

### Selection of cases and controls

Cases in which a new diagnosis of IBD was made between 2005 and 2011 were drawn from the files of this tertiary referral centre. Controls were supplied from children with IBD-like symptoms (abdominal pain, diarrhoea, poor weight gain) whose colonic biopsies had been reported as within normal limits. In all cases informed patient consent was taken before endoscopy and all six colonic sites plus the terminal ileum were biopsied in all patients. Only those cases in which an unequivocal diagnosis of UC or CD had been made by an experienced pediatric pathologist (AWHB) after multidisciplinary review of clinical and radiological findings were selected for inclusion. The control patients had no evidence of significant pathology: ancillary investigations and any biopsies taken from sites outside the colon were within normal limits, and none had been referred for repeat endoscopy due to persistent symptoms during at least two years’ follow up. The data were collected as part of an audit of histopathological reporting of gastrointestinal biopsies; no interventions were performed beyond normal clinical management and as such the study did not require ethical approval.

### Biopsy interpretation

Tissues were fixed in 4% paraformaldehyde and processed for light microscopy. Sections were cut at 6 μ thickness and stained with haematoxylin and eosin. Histological review of slides was performed using an Olympus BH-2 microscope, high power field (HPF) diameter 0.5 mm^2^, magnification × 400. For each biopsy site (caecum, ascending colon, transverse colon, descending colon, sigmoid colon, rectum) the total number of Paneth cells in the first ten well-orientated crypts was counted by an experienced histopathologist (NS or AWHB). This method was similar to that employed by Kelly et al. [19]. Three cases scored by both pathologists separately showed no significant interobserver variability. In the sections in which Paneth cells were counted, acute and chronic inflammation were scored semi-quantitatively, as in routine histopathological practice. Acute inflammation was defined as the presence of neutrophil polymorphs in surface or crypt epithelium or crypt lumina and was scored as follows: 0 = no inflammation, 1 = mild cryptitis, 2 = moderate cryptitis ± crypt abscesses, 3 = severe cryptitis ± crypt abscesses or ulceration. Chronic inflammation was defined as increased mucosal plasma cells or lymphoid aggregates and was scored: 0 = no inflammation, 1 = increased basal plasma cells, 2 = moderate increase in lamina propria plasma cells ± lymphoid aggregates, 3 = dense lamina propria plasma cells ± numerous lymphoid aggregates. Crypt architectural distortion was recorded as present if there was at least one of the following: crypt branching, cystic dilatation, atrophy or irregular orientation. Otherwise, it was recorded as absent. Eosinophil numbers within the lamina propria were assessed as described previously [20] and recorded as normal (<20 eosinophils/HPF) or hypereosinophilia (>20 eosinophils/HPF).

### Statistical analysis

Demographic information and histological features of inflammation at each anatomical site were compared between control, UC and CD groups using a chi-squared test. Paneth cell counts were compared using a two-sample Kolmogorov-Smirnov test. Correlation between Paneth cell numbers and histological features of inflammation at each site was assessed by calculating Spearman’s rank correlation coefficient.

## Results

### Clinical details and inflammation

A total of 28 cases with IBD (13 with UC and 15 with CD) and 14 controls were examined (Table 1). The commonest documented symptoms were diarrhoea, abdominal pain, rectal bleeding and weight loss. There was no statistically significant difference in symptoms between control, UC and CD groups. UC patients were more likely to have received treatment with antiinflammatories, infliximab, or immunosuppressants prior to histological diagnosis. All of the UC and CD patients had abnormal endoscopic appearances compared with only one of the controls, the histology of which showed lymphonodular hyperplasia. Extensive colitis and left-sided colitis were significantly more common in ulcerative colitis and discontinuous colitis was more common in Crohn’s disease.

**Table 1:** Symptoms and endoscopic appearances in controls and inflammatory bowel disease

		***Control***	***UC***	***CD***
N		14	13	15
Sex (M/F)		10/4	6/7	5/10
Age range (median)		6-16 (12.5)	6-15 (14)	7-16 (13)
Symptoms: n (%)	Diarrhoea	9 (64)	8 (62)	8 (53)
	Abdominal pain	9 (64)	4 (31)	7 (47)
	Rectal bleeding	6 (43)	10 (77)	6 (40)
	Weight loss	4 (29)	6 (46)	2 (13)
Treatment prior to diagnosis: n (%)	Antiinflammatories	2 (14)	7 (54)*	7 (47)
	Infliximab	0	4 (31)*	0
	Immunosuppressants	0	6 (46)†	1 (7)
Endoscopic appearance: n (%)	Abnormal	1 (7)‡	13 (100)§	15 (100)§
	Terminal ileitis	1 (7)‡	0	5 (33)
	Extensive colitis	0	7 (54)†	3 (20)
	Discontinuous colitis	1 (7)‡	1 (8)	7 (47)*
	Left-sided colitis only	0	5 (38)*	0
	Recto-sigmoid colitis only	0	2 (15)	0

*P < 0.05 (chi-squared) compared to controls.

†P < 0.005 compared to controls.

‡Histology showed lymphonodular hyperplasia.

§P < 0.00001 compared to controls.

### Paneth cell metaplasia

Paneth cells were identified in the caecum in 6 control cases, in the ascending colon in 3 cases and the transverse colon in one case, but none were present in the distal colon (descending colon, sigmoid and rectum). In patients with UC, Paneth cells were present in all but one case, and in 11 of 13 cases they were present in the distal colon. In patients with CD, Paneth cells were present in every case, and there were Paneth cells in the distal colon in 14 of 15 cases. Table 2 summarises the histological features of acute and chronic inflammation in the three groups.

**Table 2:** Features of acute and chronic inflammation in controls and inflammatory bowel disease

		***Control***	***UC***	***CD***
Highest acute inflammation score n (%)	0	14 (100)	0	0
	1	0	2 (15)	9 (60)*
	2	0	6 (46)†	2 (13)
	3	0	5 (38)*	4 (27)*
Highest chronic inflammation score n (%)	0	10 (71)	0	0
	1	4 (29)	0*	9 (60)*
	2	0	7 (54)†	4 (27)*
	3	0	6 (46)†	2 (13)
Architectural distortion present (%)		0	12 (92)‡	13 (87)‡
Eosinophils > 20/HPF (%)		1 (7)	6 (46)*	12 (80)†
PCM (%)		0	11 (85)‡	14 (93)‡

*P < 0.05 (chi-squared).

†P < 0.005.

‡P < 0.00001.

**Figure 1:**
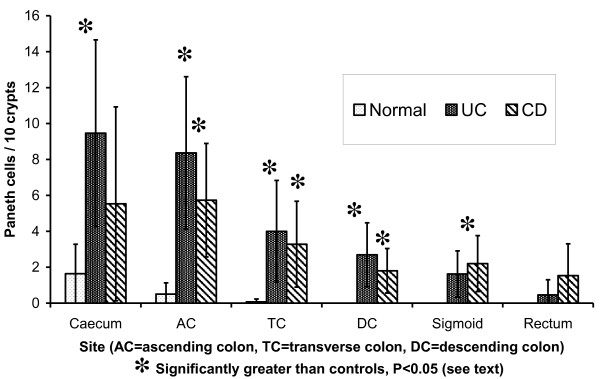
Numbers of Paneth cells (mean and 95% confidence interval) by site.

Patients with UC showed a significantly greater number of Paneth cells in the caecum (P < 0.001), ascending colon (P < 0.001), transverse colon (P < 0.006) and descending colon (P < 0.001) compared with controls (Figure 1). Patients with CD showed significantly more Paneth cells in the ascending colon (P < 0.002), transverse colon (P < 0.006), descending colon (P < 0.006) and sigmoid colon (P < 0.001) compared with controls. There were no significant differences in Paneth cell number at each anatomical site between UC and CD groups.

**Figure 2:**
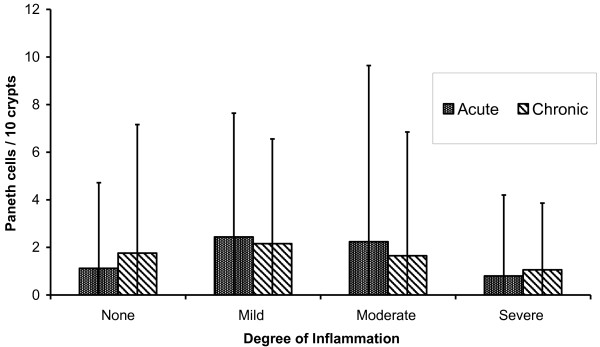
Numbers of Paneth cells (mean and 95% confidence interval) in the distal colon in IBD in relation to local inflammation.

Analysis of the features of each biopsy (n = 150) found no correlation between Paneth cell number and the degree of acute inflammation (Spearman’s rho = -0.036) or chronic inflammation (rho = 0.057), and there was no correlation with architectural distortion (rho = 0.025) or eosinophilia (rho = 0.084). In IBD patients with PCM considered separately there was no correlation between numbers of metaplastic Paneth cells at each site and scores for acute or chronic inflammation at these sites (Figure 2, rho = -0.066 and -0.034, respectively).

## Discussion

Paneth cells were found in almost half of the control group in the caecum and ascending colon, and (rarely) the transverse colon. This was comparable to the findings of the study by Tanaka et al. in adults [11], which used symptomatic patients as controls. We used a similar methodology, as it was not feasible to obtain tissue from asymptomatic children. Symptomatic controls were appropriate for testing the hypothesis that Paneth cells are a helpful diagnostic feature of newly-diagnosed paediatric IBD, since this diagnosis is only considered in symptomatic children. There was no evidence of significant pathology in our control group: four children had a focal non-specific mild increase in chronic inflammatory cells in the lamina propria and the remaining ten had no histological abnormality. Their symptoms were most likely to have been due to irritable bowel syndrome. While we cannot entirely exclude IBD or other colonic pathology having developed subsequently, this is unlikely as re-referral for repeat colonoscopy is the standard procedure if IBD-like symptoms persist, and no patient in the control group had been re-referred after a minimum of two years follow up. We conclude that Paneth cells are present in the proximal colon of children in the absence of significant disease. It is possible that altered bowel habit or other physiological changes affected Paneth cell numbers in the control group, so these findings are not necessarily applicable to asymptomatic children. Treatment prior to referral may also have modified Paneth cell numbers in both control and IBD groups.

Despite these potential confounding factors, there were clear differences between controls and IBD patients. Paneth cell numbers were significantly higher in the proximal colon in IBD, which suggests that Paneth cell hyperplasia occurs here. More than ten Paneth cells per ten crypts at any site was seen only in IBD. PCM in the distal colon was present in the great majority of patients with IBD. Paneth cell numbers were significantly increased in IBD at all sites except the rectosigmoid in UC and the caecum and rectum in CD, and even in these areas Paneth cell counts were somewhat higher, so it is likely that statistical significance would be achieved with a larger series. One case of UC had no Paneth cells, possibly due to their being patchily distributed, though some colons may have no Paneth cells at all [11].

Paneth cells are implicated in both the pathogenesis of and the response to IBD. Decreased Paneth cell activity in the terminal ileum in a subset of patients with ileal CD who have mutations of the susceptibility gene *NOD2* may be an early or initiating factor in their disease [6,7,21]. Alpha defensin expression, an indicator of Paneth cell activity, is very low in the colon compared with the normal terminal ileum, but it is raised in patients with IBD, including children with colonic Crohn’s disease, possibly due to the presence of hyperplastic or metaplastic Paneth cells [7]. Paneth cell hyperplasia and metaplasia may be a local response to inflammatory changes in IBD, or a reaction to pan-colonic changes such as altered bacterial flora [11]. Increased colonic Paneth cells in IBD may be part of an adaptive reaction in which alpha defensins constitute a protective barrier that helps prevent secondary mucosal infection in already inflamed bowel [22]. Alternatively, it has been suggested that PCM in the colon may exacerbate or even initiate IBD there [23]. The interplay of factors is complex and the role of Paneth cells in the pathogenesis of colonic IBD remains unclear.

Kumarasinghe et al. [24] identified PCM in 2 of 25 (8%) cases of adult Crohn’s disease at initial presentation but not in 5 patients with tuberculosis or diverticular disease, which led them to suggest that PCM might be an aid to the initial diagnosis of Crohn’s disease. The present study shows that PCM is a feature of newly-diagnosed IBD in children and is present in a high proportion of cases at diagnosis: 85% of UC and 93% of CD patients. In neither adults nor children is PCM specific to IBD – for example, it has been demonstrated in chronic diverticulitis [25] and after necrotizing enterocolitis [15] – but it may be be useful as an adjunct to the diagnosis of IBD in biopsies from symptomatic children. Tanaka et al. [11] found a correlation between PCM and crypt architectural distortion in adults and considered PCM a feature of chronic IBD. In our paediatric patients we found no correlation between PCM and histological features of chronicity (increased lymphocytes, crypt architectural distortion), or between PCM and acute inflammation or eosinophilia. Since Paneth cells are renewed approximately every 30 days, it is not possible to say from our series whether PCM is present in IBD *ab initio*, since the time between onset of symptoms and referral often exceeded this. For practical purposes, however, since PCM is seen in a high proportion of cases of paediatric IBD at the time of diagnosis it is not specific for established disease. A follow-up study of Paneth cell numbers in a paediatric population with longstanding IBD is planned to determine whether their numbers and distribution change as disease progresses.

## Conclusions

PCM is present in most cases of paediatric IBD at the time of diagnosis. It does not correlate with histological features of chronic inflammation and does not appear to be a sign of chronicity. We recommend that the presence of PCM (Paneth cells in the distal colon) or hyperplasia (>10 Paneth cells per 10 crypts in the proximal colon) should be reported in paediatric colonoscopy series as they are features suggestive though not diagnostic of IBD.

## Competing interests

The authors declare that they have no competing interests.

## Authors’ contributions

NS and AWHB performed the histological examination, analysed the data and drafted the manuscript. MF and EK performed the endoscopies and provided clinical interpretation. AP, NS and AWHB designed the study. All authors read and approved the final manuscript.

## Pre-publication history

The pre-publication history for this paper can be accessed here:

http://www.biomedcentral.com/1471-230X/14/93/prepub
